# Supplementary material for: Notch Signaling Regulates Mouse Perivascular Adipose Tissue Function via Mitochondrial Pathways
Source: Genes (Basel). 2023 Oct 20;14(10):1964. doi: 10.3390/genes14101964 (PMC10606454; doi:10.3390/genes14101964)
Supplement: Supplementary file 1 [file genes-14-01964-s001.zip › genes-2624466-supplementary.pdf]

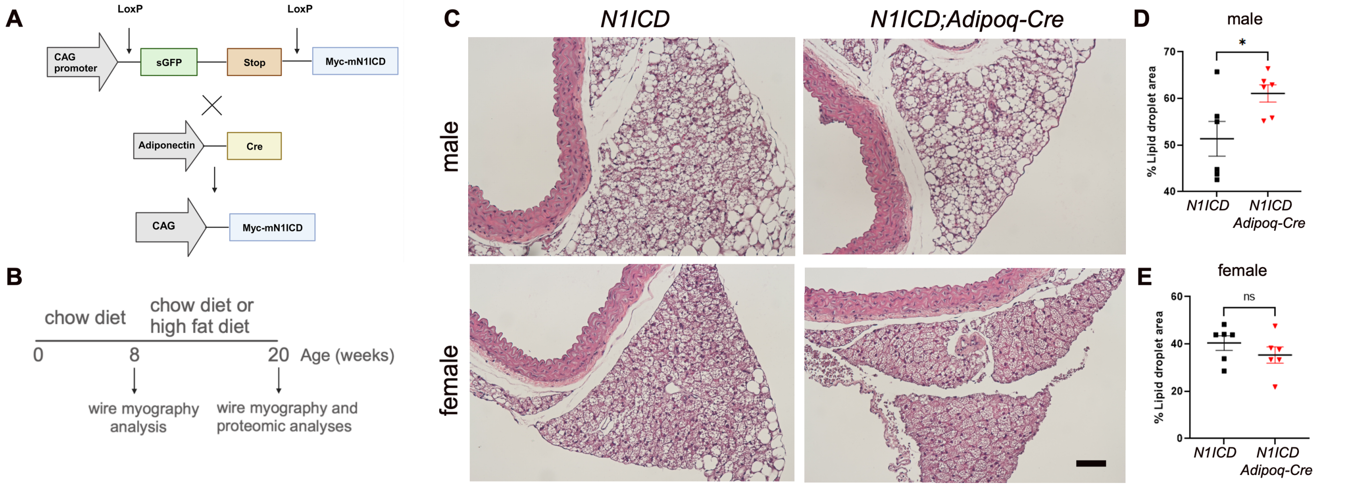

Supplemental Figure 1. Notch activation in adipose tissue regulates lipid accumulation in PVAT in a sex-dependent manner. (**A**) Schematic diagram shows the strategy of crossing the *N1ICD* strain with the *AdipoqCre* strain to activate Notch signaling in adipocytes. *N1ICD* positive / Cre negative mice are referred to as *N1ICD,* and double positive mice are referred to as *N1ICD;AdipoqCre.* (**B**) Experimental design to analyze the effects of a regular chow or high fat diet and age in vascular reactivity and protein profiles. (**C**) PVAT and adjacent thoracic aorta was collected from 20-week-old *N1ICD* and *N1ICD;Adipoq-Cre* mice fed a chow diet. Tissues were processed for paraffin embedding, sectioning and hematoxylin/eosin staining. In male mice (top panels), Notch activation in adipose tissue led to adipocyte hypertrophy with increased lipid deposition in PVAT. This difference was not observed in the PVAT of female mice (lower panels). Scale bar= 100□m. (**D-E**) Quantifications were performed on n=6 mice for each genotype and sex. Graphed are means ± SEM. Statistical analysis was performed using Student's t-test. *p<0.05 and ns = not significant.

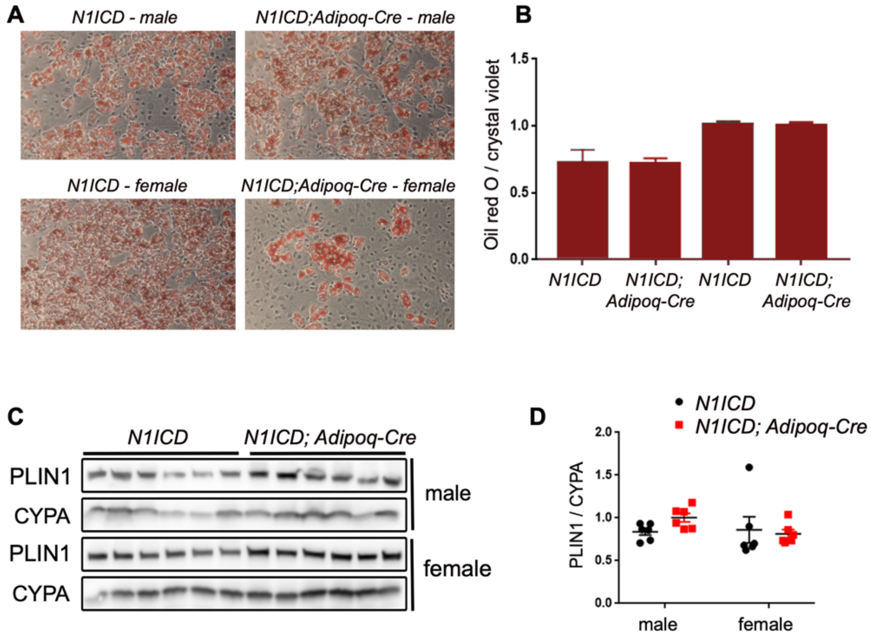

Supplemental Figure 2. Notch activation in adipocytes does not affect differentiation or lipid accumulation. (**A**)SVF was derived from male or female mice of the indicated genotypes and differentiated in vitro for seven days. Cells were fixed and stained with Oil red O to quantify lipid. (**B**) Quantification of Oil red O staining normalized to crystal violet staining of cell nuclei. Of note, the *N1ICD;Adipoq-Cre* cells from female mice at the end of the experiment were at a lower cell density compared to cells derived from *N1ICD* females. (**C**) Immunoblotting for perilipin 1 (PLIN1) and Cyclophilin A (CYPA, housekeeping protein) in the differentiated PVAT-SVF from male or female mice with the indicated genotypes. (**D**) Quantification of immunoblotting in (C) showed similar PLIN1 levels between groups.
